# Bridging the gap: effects of simulation-based OB/GYN training on skills and self-perception in final-year medical students

**DOI:** 10.3389/fmed.2025.1716282

**Published:** 2025-12-16

**Authors:** Jana Adams, Christoph Stosch, Michael Mallmann, Niels-Benjamin Adams, Sebastian Ludwig

**Affiliations:** 1 Department of Gynecology and Obstetrics, Faculty of Medicine and University Hospital Cologne, University of Cologne, Cologne, Germany; 2 Cologne Interprofessional Skills-Lab and Simulation Centre, Faculty of Medicine and University Hospital Cologne, University of Cologne, Cologne, Germany; 3 Department of Gynecology and Obstetrics, Faculty of Medicine, Public Hospital of Cologne, University of Cologne, Cologne, Germany; 4 Department of Anesthesiology and Intensive Care Medicine, Faculty of Medicine and University Hospital Cologne, University of Cologne, Cologne, Germany; 5 Department of Gynecology, Faculty of Medicine and University Hospital Cologne, University of Cologne, Cologne, Germany

**Keywords:** medical students, medical education, practical skills, curriculum, simulation, OB/GYN, self-assessment alignment, objective assessment

## Abstract

**Background:**

Structured, simulation-based teaching is increasingly recognized as essential for clinical competence development. In Germany, however, practical training during the final year (Praktisches Jahr, PJ) remains inconsistently structured, particularly in obstetrics and gynecology (OB/GYN).

**Methods:**

A quasi-experimental pre–post study compared final-year students participating in *ready4gyn*, a structured OB/GYN simulation curriculum, with a comparison group from affiliated hospitals. The intervention group completed pre- and postrotation self-assessment (19 items) and objective assessments, (14 items) on 7-point Likert scales, whereas the comparison group completed postrotation self-assessment only. Non-parametric tests (Wilcoxon, Mann–Whitney U, Kruskal–Wallis) were used for analysis.

**Results:**

Among the intervention group (*N* = 65), self-assessed competence and objectively measured skills increased significantly from median 50 to 54, and objectively measured skills from 27 to 47 (both *p* < 0.001). The discrepancy between self-perceived and actual performance decreased to near zero (*p* < 0.001). When comparing groups at the end of the rotation, intervention students reported higher self-assessed competence than students in usual rotations.

**Conclusion:**

Participation in the *ready4gyn* curriculum was associated with substantial pre–post gains in perceived and objectively measured competence within the intervention group and with higher end-of-rotation self-assessed competence compared with students in standard clinical rotations. Because only the intervention group received objective and longitudinal assessments, conclusions regarding between-group effects are limited to self-assessment outcomes. The findings support integration of structured simulation-based training into undergraduate OB/GYN education.

## 1 Introduction

Medical education is a fundamental component of preparing future healthcare professionals to deliver high-quality patient care. In Germany, the medical curriculum is designed to integrate theoretical knowledge, skills and attitudes to develop competencies.

A key element of medical and clinical practice is the hands-on performance of specific examination or treatment procedures. In partially surgical disciplines such as obstetrics and gynecology (OB/GYN), clinical examination skills are of particular importance. Both the Masterplan Medical Studies 2020 (1), a national reform initiative introduced by the German government to modernize medical education, and the new medical licensing regulations place special emphasis on the early acquisition of practical skills during medical school.

It has already been shown that learning and practicing on models or in high-quality simulations can provide an ideal learning environment (2–4). Students can freely experiment with techniques and procedures and there is room for making mistakes, these are even encouraged, as they allow students to identify the sources of errors through feedback and constructive criticism, helping them to avoid similar issues in real clinical settings (5). Reflection as a metacognitive competence (in a training session as formative evaluation, feedback e.g., debriefing) seems to be the key for learning experiences in education (6).

While practical skills should ideally be acquired during undergraduate medical training, comprehensive and specialty-specific examination techniques, such as those required in gynecology, are often difficult to achieve within the core curriculum (7).

The Praktisches Jahr (PJ), or practical year, serves as the final year of medical education in Germany and offers students the opportunity to gain direct experience in patient care.

This year is typically divided into three 16-week full-time rotations, where OB/GYN can be chosen as an elective subject in addition to the mandatory rotations in internal medicine and general surgery.

It is generally assumed that medical students enter the final year (Praktisches Jahr, PJ) with a solid foundation of practical skills. The PJ is typically viewed as a phase for applying these previously acquired competencies in real clinical settings, further developing routine and confidence in daily practice. Re-teaching of basic clinical skills usually does not occur during this phase. In this context, the concept of Entrustable Professional Activities (EPAs) has gained relevance. An EPA describes a core medical task in terms of scope and depth, as it should be performed by medical students at the end of their final year under a defined level of supervision. It is part of a set of 12 core medical activities that delineate the expected outcomes for the completion of undergraduate medical education and the transition to postgraduate training. These EPAs were developed within the framework of the National Competence-Based Cataloge of Learning Objectives for Medicine (Nationaler Kompetenzbasierter Lernzielkatalog Medizin, in Germany) (8).

Previous studies have shown that final-year medical students often display deficits in clinical competencies in OB/GYN, especially when assessed through Objective Structured Clinical Examinations (OSCEs), which more accurately reflect real-life clinical performance than written exams (9). This highlights the need for structured, hands-on training formats.

At the same time, research suggests that students’ self-assessments of their clinical skills are often inaccurate, being influenced by confidence, previous experience and the prevailing feedback culture (10, 11). While self-perception plays an important role in reflective practice, it does not always align with actual performance. Sole reliance on self-assessment can therefore be misleading (12).

In this context, the OB/GYN department of the University Hospital of Cologne implemented a structured practical training program *ready4gyn* for students in their clinical OB/GYN rotation. What sets this initiative apart is its systematic scientific evaluation: this study not only introduces a practice-oriented curriculum within the final year of medical school, but also rigorously examines its educational impact. By combining self-assessment and objective evaluation, it enables the identification of discrepancies between perceived and actual competence, thus promoting self-awareness, supporting professional development, and offering valuable insight into student satisfaction and learning outcomes.

## 2 Materials and methods

Ethical approval was obtained from the Ethics Committee of the Faculty of Medicine of the University of Cologne on 11/11/21 (Ref. No. 22-1341).

This study followed a quasi-experimental, non-randomized pre–post design comparing an intervention group with a non-equivalent control group. This study was designed and reported in accordance with the STROBE statement for observational studies.

**FIGURE 1:**
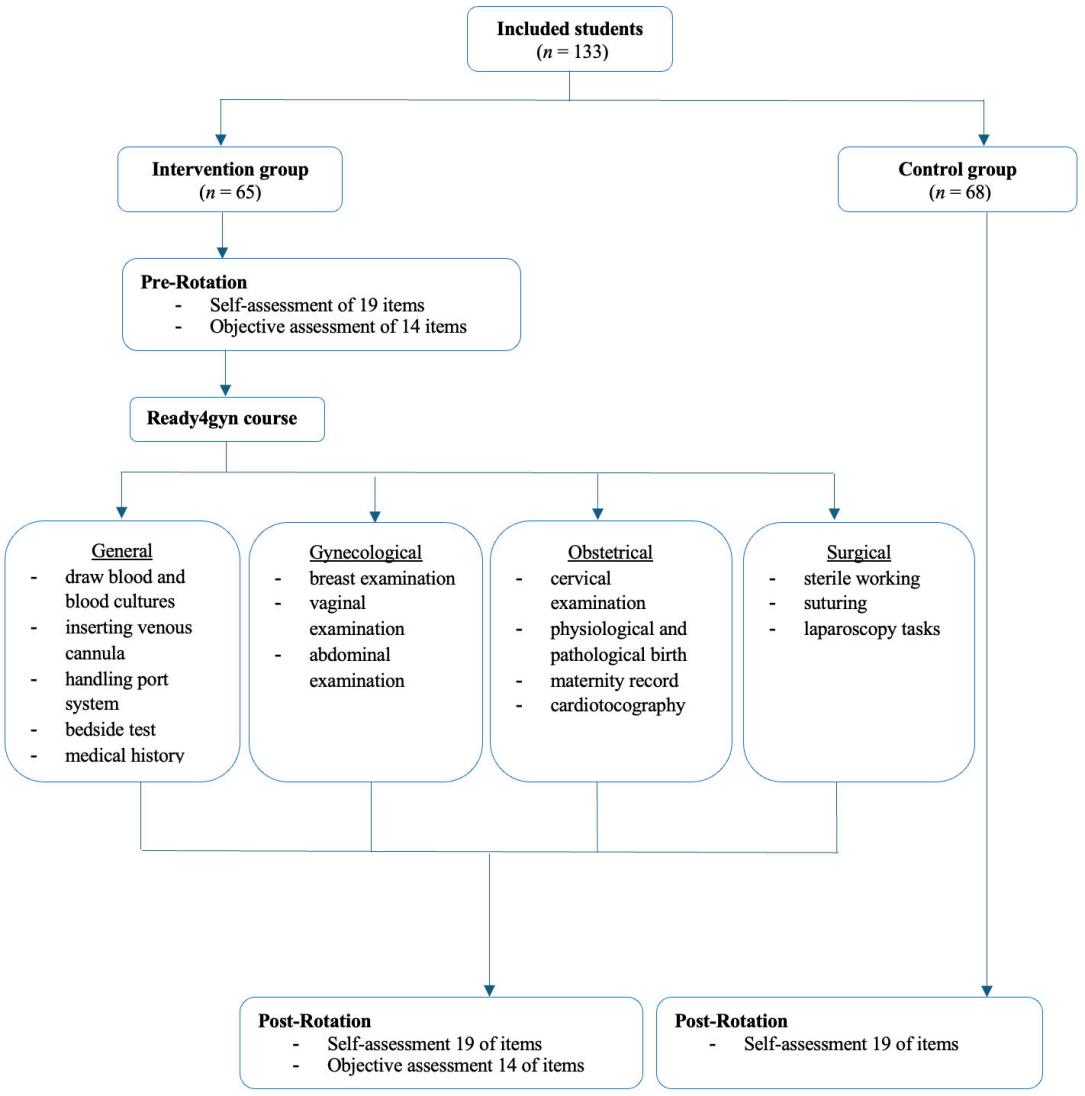
Study design for the curriculum of the practical course ready4gyn.

A structured practical curriculum titled *ready4gyn* was introduced in 2022 as part of the elective OB/GYN rotation during the final practical year of medical school at the University Hospital of Cologne. Every 4 weeks, students participated in a 2-hour practical session focusing on general, gynecological-specific, obstetric-specific and surgical-specific skills in four sessions throughout the OB/GYN rotation (see Figure 1).

During the general session, basic universal clinical tasks such as drawing blood, collecting blood cultures, inserting a venous cannula, and handling a port system were taught using phantom models [ACF Pad Venipuncture (Item No. 00140), Limbs & Things Ltd., Bristol, United Kingdom; Chester Chest™ (Item No. VT2400), Laerdal Medical GmbH, Puchheim, Germany]. Bedside tests were performed and interpreted using real blood. Medical history taking was practiced interactively through two-person simulations. In the gynecological session, breast and vaginal examinations were trained using phantom models [Advanced Breast Examination Trainer (Item No. 40201), Limbs & Things Ltd., Bristol, United Kingdom; EVA Pelvic Teaching Model (Item No. 1900), Nasco Healthcare, Fort Atkinson, WI, United States]. After practicing physiological findings, pathological findings such as suspicious breast lumps and abnormal cervical changes were demonstrated. In the obstetrical part, cervical examinations were practiced using a phantom model [Cervical Dilation and Effacement Simulator (Item No. R10922), Erler-Zimmer GmbH & Co., KG, Lauf an der Pegnitz, Germany], covering a range of findings from an unripe cervix to a cervical dilation of 9 cm. Both physiological and pathological birth mechanics were demonstrated using a phantom model [PROMPT Birthing Simulator (Item No. 80100), Limbs & Things Ltd., Bristol, United Kingdom]. CTG interpretation was taught by reviewing interpretation criteria and applying them to real-life examples, including both normal and abnormal patterns. Maternity record interpretation was practiced using three different cases. The surgical component included sterile working techniques (preparing a sterile field, putting on sterile gloves), suturing techniques (interrupted, subcutaneous, and intracutaneous sutures) using a phantom model [Suture Tutor Trainee Kit (Item No. 90021), Limbs & Things Ltd., Bristol, United Kingdom], as well as a laparoscopy course containing the Fundamentals of Laparoscopic Surgery (13) tasks peg transfer and precision cutting, using a laparoscopy trainer (eosim, eoSurgical Ltd., Edinburgh United Kingdom).

The course and data collection were conducted in the Skills Lab of the Faculty of Medicine of the University of Cologne, KISS (Kölner Interprofessionelles Skills Lab und Simulationszentrum).

All final-year medical students in Cologne – both in the intervention and control groups – followed the same set of learning objectives for the OB/GYN rotation. These objectives are documented in a logbook and are aligned with the EPAs, which define key competencies to be acquired by the end of the PJ. The learning objectives are based on the PJ curriculum for gynecology developed by the German Society of Gynecology and Obstetrics (Deutsche Gesellschaft für Gynäkologie und Geburtshilfe, DGGG).

In the intervention group, all 65 final-year medical students completing their OB/GYN rotation at the university hospital were invited to participate and all consented, resulting in a complete data set without dropouts or missing values. For the comparison group, recruitment was conducted via an email distribution list addressed to all final-year students completing their OB/GYN rotation at affiliated teaching hospitals during the study period. Because this recruitment pathway did not allow tracking of the total number of students reached, the exact denominator of invited students is unknown. A total of 68 students responded and provided complete postrotation self-assessments. No exclusions were made in either group, and no missing data occurred in the primary variables; thus, no imputation or listwise deletion procedures were required.

For the primary outcomes, effect sizes for non-parametric tests were calculated using the rank-biserial correlation (r). Given the large number of item-level comparisons, these analyses were considered exploratory. Therefore, no formal correction for multiple testing was applied; however, interpretation focused on the consistent pattern of highly significant findings and large effect sizes in the aggregated scores.

A statistical power analysis was performed using G*Power to determine the required sample size. With an alpha of 0.05 and a power of 0.8, the required sample size for detecting a medium effect (Cohen’s d = 0.6) in intergroup comparisons with a two-tailed *t*-test for independent samples was determined to be at least *N* = 90 participants.

Although the analyses were ultimately conducted using non-parametric tests due to the ordinal nature of the data, the *t*-test-based power estimation remains appropriate. The *t*-test and Mann–Whitney U test have comparable power under most conditions, and sample size requirements for non-parametric tests are typically similar or slightly lower. Thus, the estimated sample size was sufficient for the statistical procedures applied. The achieved sample size (*N* = 133) exceeded the estimated minimum of 90 participants, confirming adequate statistical power for all primary comparisons.

Inclusion criteria were as follows. All medical students completing their OB/GYN rotation at the University Hospital of Cologne between 2022 and 2023 were asked to participate voluntarily as the intervention group. Students rotating at affiliated teaching hospitals of the University of Cologne (not at the University Hospital itself) were included as the control group. No exclusion criteria were defined.

Students at the University Hospital of Cologne were asked to complete a self-assessment at the beginning and end of their OB/GYN rotation covering 19 practical skills (8 general and 10 gynecology-specific and one rating readiness for clinical work). 14 of these skills were also objectively assessed by experts at both time points (see Table 1). Each item was rated using a 7-point Likert scale in response to the question “How confident do you feel performing the following skill?” (1 = very uncertain, 2 = uncertain, 3 = somewhat uncertain, 4 = neutral, 5 = somewhat certain, 6 = certain, 7 = very certain). An evaluation was conducted using the 7-point Likert scale as well (1 = totally disagree, 2 = disagree, 3 = somewhat disagree, 4 = neutral, 5 = somewhat agree, 6 = agree, 7 = totally agree).

**TABLE 1:** Items assessed through self- and objective evaluations at the start and end of the rotation.

	Item	Self-assessment	Objective assessment
General skills	Draw blood	X	X
	Insert venous cannula	X	X
	Draw blood cultures	X	X
	Handling a port system	X	X
	Bedside test	X	X
	Taking medical history	X	
	Abdominal examination	X	X
	Working sterile	X	X
Gynecological skills	Vaginal examination	X	X
	Breast examination	X	X
	Sonography	X	
	Suturing	X	X
	Laparoscopy	X	
	Cervical examination	X	X
	Maternity record	X	
	Cardiotocography	X	X
	Physiological birth	X	X
	Pathological birth	X	X
	Ready for the job	X	
Evaluation	Professional supervision	X	
	Time frame appropriate	X	
	Opportunity to ask questions	X	
	Learning objectives clearly structured	X	
	Training content applicable to clinical practice	X	
	High learning gain	X	
	Interest stimulated	X	
	Overall evaluation of the ready4gyn course	X	

Additionally, students completing their OB/GYN rotation at teaching hospitals (without access to the practical curriculum) were asked to complete the same self-assessment at the end of their rotation, serving as a control group.

Self-assessment was administered digitally using LimeSurvey Community Edition (version 5.6.68 + 240625; LimeSurvey GmbH, Hamburg, Germany). Objective assessment was conducted in a format similar to an Objective Structured Clinical Examination (OSCE). Two trained raters conducted the objective assessments. Both raters were clinicians experienced in OB/GYN teaching and underwent a calibration session prior to data collection to ensure consistent scoring. The assessment checklists were developed based on established OB/GYN learning objectives and were reviewed by two senior specialists for content validity. Before implementation, the checklist items were piloted with a small cohort of students (*N* = 5) to confirm clarity and feasibility. Not all skills were evaluated objectively due to logistical constraints, e.g., ultrasound examinations or medical history taking were difficult to standardize and therefore excluded from objective evaluation.

All assessed items were taught during the practical course, with the exception of ultrasound skills, which were covered separately through patient-based teaching due to the lack of simulation tools.

An evaluation of the course *ready4gyn* was also conducted. Statistical analyses were performed using IBM SPSS Statistics (version 30.0, Armonk, NY, USA). To examine changes in assessment scores within the same group from pre- to post-rotation, the Wilcoxon signed-rank test was used. To explore the association between self-assessed and objectively measured competence, Spearman’s rank correlation coefficients (ρ) were calculated for overall self- and objective scores before and after the intervention. Comparisons of self-assessment scores between students from different hospitals (intervention vs. control group) were conducted using the Mann–Whitney U test. To analyze potential effects of rotation order on assessment results (pre- and postrotation), the Kruskal–Wallis test was applied to compare three independent groups. To enhance readability and clarity, only aggregated results (median, interquartile range, and *p*-values) are reported in the main tables. For non-parametric tests (Wilcoxon signed-rank, Mann–Whitney U, and Kruskal–Wallis), test statistics (*Z*- and *U*-values, ranks, and ties) were omitted as they do not add interpretive value beyond the reported significance levels. Detailed descriptive data and complete test statistics are available in the Supplementary material.

Internal consistency of the 19-item self-assessment instrument was examined using Cronbach’s alpha (reported in the Results section) to evaluate the reliability of the scale.

Potential sources of bias, such as self-selection and assessor subjectivity, were minimized through standardized rater calibration, consistent learning objectives across sites, and uniform assessment procedures. No missing data occurred for the main variables; incomplete cases were excluded listwise.

## 3 Results

### 3.1 Demographic data

A total of 133 students were included in the final sample: 65 (48.9%) students in the intervention group (university hospital) who underwent practical training and 68 (51.1%) students in the control group (teaching hospitals). Participants demographic data are presented in Table 2.

**TABLE 2:** Participants demographic data.

	Total *N* = 133	University hospital *N* = 65	Teaching hospital *N* = 68
Age in years	26 [25.5–27]	26 [25.5–27]	26 [26–26]
**Gender**			
Male	13 (9.8%)	7 (10.8%)	6 (8.8%)
Female	120 (90.2%)	58 (89.2%)	62 (91.2%)
**Clinical rotation**			
1^st^	34 (25.6%)	15 (23.1%)	19 (27.9%)
2^nd^	53 (39.8%)	29 (44.6%)	24 (35.3%)
3^rd^	46 (34.6%)	21 (32.3%)	25 (36.8%)
**German language skills**			
Mother tongue	121 (91.0%)	58 (89.2%)	63 (92.6%)
C (proficient)	9 (6.8%)	4 (6.2%)	5 (7.4%)
B (independent)	3 (2.3%)	3 (4.6%)	0 (0%)
**Mother tongue**			
German	114 (85.7%)	55 (84.6%)	59 (86.8%)
Turkish	10 (7.5%)	5 (7.7%)	5 (7.4%)
Russian	3 (2.3%)	2 (3.1%)	1 (1.5%)
Arabic	3 (2.3%)	1 (1.5%)	2 (2.9%)
Romanian	1 (0.8%)	1 (1.5%)	0 (0%)
Persian	1 (0.8%)	1 (1.5%)	0 (0%)
Spanish	1 (0.8%)	0 (0%)	1 (1.5%)

Results in total and subdivided into location of the final year OB/GYN rotation. Quantitative data are presented as median and interquartile range [Q25–Q75] and qualitative data as frequency counts (percentage).

The median overall age was 26 years [IQR 25.5 - 27], ranging from 24 to 34 years.

The vast majority were female (*N* = 120, 90.2%) while 13 students (9.8%) were male. Most students were in their second (*N* = 53, 39.8%) or third (*N* = 46, 34.6%) clinical rotation, compared with 34 students (25.6%) in their first rotation.

Regarding native language, 114 students (85.7%) reported German, 10 (7.5%) Turkish and 3 (2.3%) Russian and Arabic each; one student (0.8%) reported Romanian, Persian and Spanish each.

German language proficiency was C-level (proficient user) for 9 students (6.8%) and B-level (independent user) for 3 students (2.3%); the remaining 121 students (91%) spoke German at a native level.

Demographic characteristics were similar between the university hospital and teaching hospital groups (see Table 2), except for rotation order: The majority (*N* = 29, 44.6%) of university hospital students were in their second rotation, whereas at teaching hospitals, most students (*N* = 25, 36.8%) were in their third rotation.

### 3.2 Self-assessment and objective assessment (intervention group)

Changes in perceived and objectively measured clinical competence were assessed by comparing pre- and postrotation self-assessment scores using the Wilcoxon signed-rank test (paired, ordinal data).

Following the *ready4gyn* course, students in the intervention group demonstrated significant improvements in both perceived (19 items) and objectively assessed (14 items) clinical skills, as measured by the Wilcoxon signed-rank test (*p* < 0.001 for all comparisons; see Table 3).

**TABLE 3:** Wilcoxon signed-rank test results for changes in pre-/post-intervention comparison of self- and objectively assessed skills.

Skill	Self-assessment			Objective assessment		
	Pre-rotation *N* = 65 Median [IQR]	Post-rotation *N* = 65 Median [IQR]	*p*-value	Pre-rotation *N* = 65 Median [IQR]	Post-rotation *N* = 65 Median [IQR]	*p*-value
Draw blood	6 [6–7]	7 [7–7]	<0.001	4 [3–5]	7 [7–7]	<0.001
Venous cannula	6 [5–7]	7 [7–7]	<0.001	4 [2–5]	7 [7–7]	<0.001
Blood cultures	5 [4–6]	7 [7–7]	<0.001	3 [2–4]	7 [7–7]	<0.001
Port system	5 [3.5–6]	7 [6–7]	<0.001	3 [2–4]	7 [6–7]	<0.001
Bedside test	5 [4–6]	7 [6–7]	<0.001	4 [2–4]	7 [6–7]	<0.001
Medical history	6 [5–6]	7 [6–7]	<0.001	4 [3–5]	6 [6–7]	<0.001
Abdominal examination	6 [5–6]	7 [6–7]	<0.001	n.a.	n.a.	n.a.
Steril working	5 [5–6]	6 [6–7]	<0.001	3 [2–4]	6 [6–7]	<0.001
General skills overall	39 [33–43]	47 [45–49]	<0.001	27 [16–32]	47 [46–48]	<0.001
Vaginal examination	3 [2–3.5]	5 [5–5]	<0.001	1 [1–1]	4 [4–5]	<0.001
Breast examination	5 [5–6]	6 [6–7]	<0.001	3 [2–4]	6 [6–7]	<0.001
Sonography	4 [4–5]	5 [5–6]	<0.001	n.a.	n.a.	n.a.
Suturing	5 [4–6]	6 [5.5–7]	<0.001	3 [2–4]	6 [6–6]	<0.001
Laparoscopy	4 [3–5]	5 [4–6]	<0.001	n.a.	n.a.	n.a.
Cervical examination	2 [1–3]	4 [4–4.5]	<0.001	1 [1–1]	4 [4–5]	<0.001
Maternity record	4 [4–5]	7 [6–7]	<0.001	n.a.	n.a.	n.a.
CTG	5 [4–5]	6 [5–6]	<0.001	2 [1–2]	0 [0–0]	<0.001
Physiological birth	5 [4–5]	6 [5–7]	<0.001	1 [1–2]	0 [0–0]	<0.001
Pathological birth	4 [3–5]	6 [5–6]	<0.001	2 [1–2]	0 [0–0]	<0.001
OB/GYN skills overall	41 [35–46]	56 [49.5–59]	<0.001	10 [8–12.5]	0 [−1.5 to 1]	<0.001
Ready for the job	5 [4–5]	6 [6–7]	<0.001	n.a.	n.a.	n.a.

Results are reported as median, interquartile range [Q25–Q75] and significance level; n.a. indicating not assessed.

**FIGURE 2:**
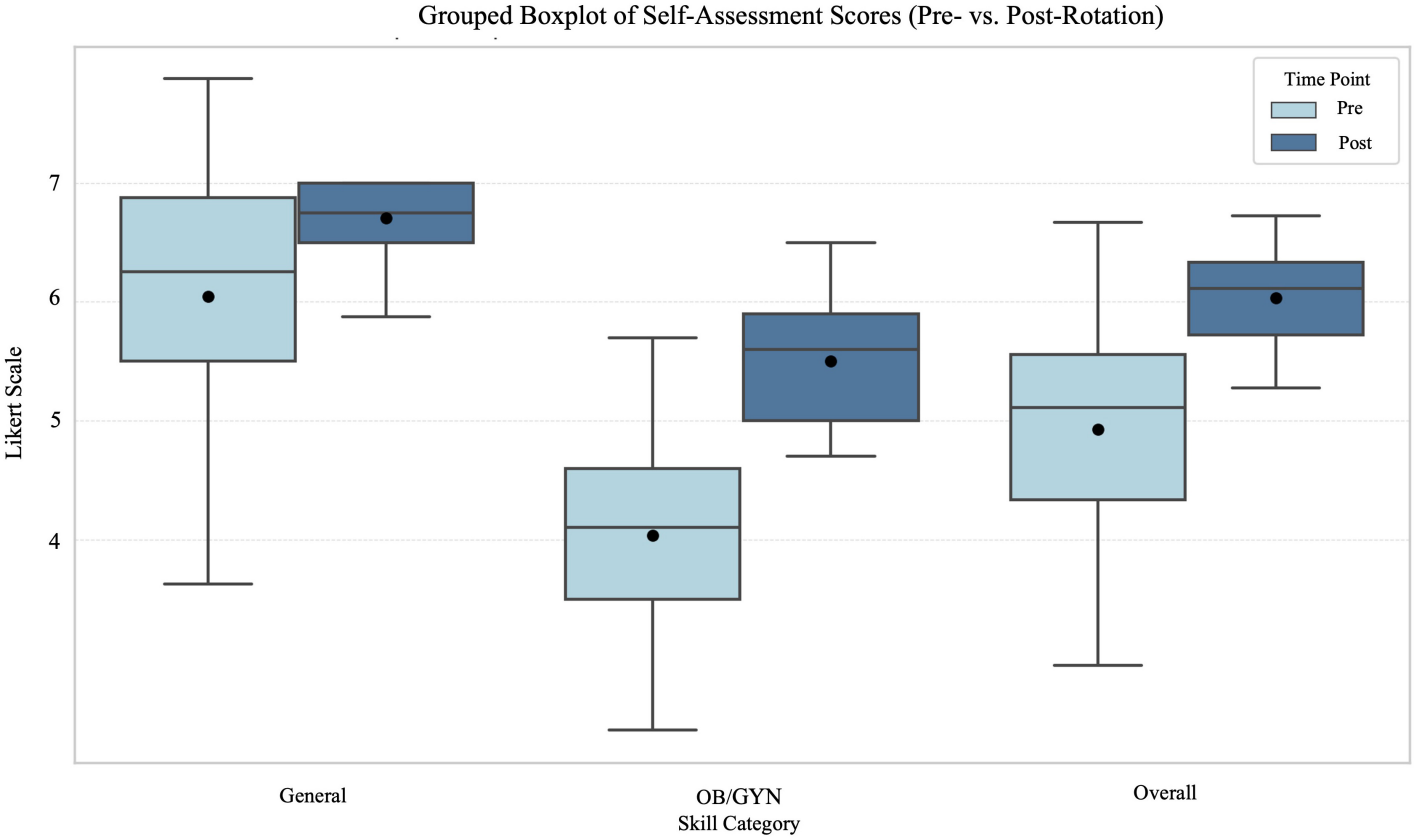
Grouped boxplot showing self-assessment scores for general, OB/GYN and overall skills before and after the OB/GYN rotation showing IQR, median, mean scores. Likert scale ranged from 1 (very uncertain) to 7 (very certain).

The Wilcoxon signed-rank test revealed a significant increase in students’ self-assessed competence in overall score for general skills (formed from the summed scores of the eight items that were also objectively assessed) with the median score rising from 50 [IQR 42.5–55] pre-intervention to 54 [IQR 51.5–56] post-intervention (*Z* = [5.07], *p* < 0.001), see Figure 2. The corresponding effect size for this change was large (r ≈ 0.63). The number of positive ranks (i.e., students reporting improvement) was 49, while negative ranks numbered 13, indicating a consistent pattern of improvement. Overall score for gynecological skills (10 items) showed a median score rising from 41 [IQR 35–46] pre-intervention to 56 [IQR 49.5–59] post-intervention (*Z* = [7.02], *p* < 0.001). All 65 students showed positive ranks (i.e., students reporting improvement), while none showed a negative difference, suggesting a robust and consistent enhancement in perceived competence. The effect size for this change was also large (r ≈ 0.87).

Rating ready for the job, median score went from 5 [IQR 4–5] to 6 [IQR 6–7], *Z* = [7.18] and *p* < 0.001) with 65 positive and no negative ranks.

This pattern held across all 19 items, with most post-intervention median scores reaching the maximum of 7. In particular, the items drawing blood and inserting a venous cannula received perfect rating (7) from every student postrotation.

**FIGURE 3:**
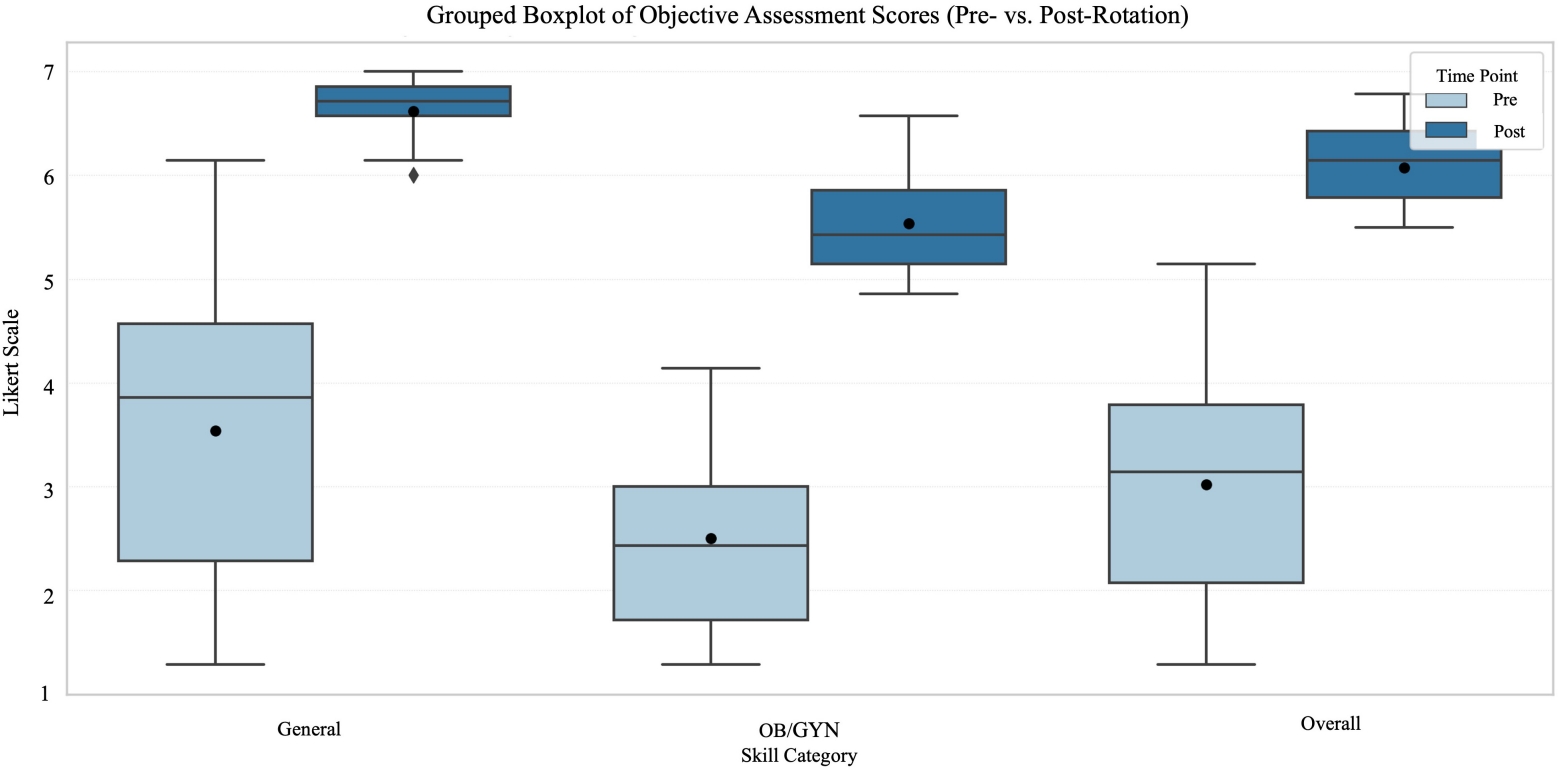
Grouped boxplot showing objective assessment scores for general, OB/GYN, and overall skills before and after the OB/GYN rotation showing IQR, median, mean scores. Likert scale ranged from 1 (very uncertain) to 7 (very certain).

A significant increase in objectively assessed clinical competences in overall score for general skills (formed from the summed scores of the seven items that were also objectively assessed) with the median score rising from 27 [IQR 16–32] pre-intervention to 47 [IQR 46–48] post-intervention (*Z* = [7.01], *p* < 0.001), see Figure 3. The effect size indicated a very large improvement (r ≈ 0.87). Overall score for gynecological skills (7 items) showed a median score rising from 17 [IQR 12–21] pre-interventional to 38 [IQR 36–41] post-intervention (*Z* = [7.02], *p* < 0.001). The corresponding effect size was also large (r ≈ 0.87). For both, the general and gynecological objective total scores, all 65 students showed positive ranks and none showed a negative difference.

The 19-item self-assessment scale showed excellent internal consistency (Cronbach’s alpha = 0.959), indicating high reliability.

### 3.3 Alignment between self- and objective assessment

The discrepancy between self- and objective assessments (self-score minus objective score) was calculated for each of the 14 items and compared pre- vs. postrotation via Wilcoxon signed-rank tests (paired and ordinal data). There was a statistically significant reduction in discrepancies across all items (all *p* < 0.001; see Table 4). Additional detailed statistical results, including full test statistics and item-level data for all non-parametric analyses, are provided in the Supplementary Table 1.

**TABLE 4:** Wilcoxon signed-rank test results for changes in pre-/post-intervention comparison.

Skill	Pre-rotation *N* = 65 Median [IQR]	Post-rotation *N* = 65 Median [IQR]	*p*-value
Draw blood	2 [1–3]	0 [0–0]	<0.001
Venous cannula	2 [2–2]	0 [0–0]	<0.001
Blood cultures	2 [1–2]	0 [0–0]	<0.001
Port system	2 [1–3]	0 [0–0.5]	<0.001
Bedside test	2 [1–2]	0 [0–0]	<0.001
Abdominal examination	1 [1–2]	0 [0–0]	<0.001
Steril working	2 [1–3]	0 [0–0]	<0.001
General skills overall	14 [9–15]	0 [0–1.5]	<0.001
Vaginal examination	2 [1–3]	0 [0–1]	<0.001
Breast examination	2 [1–3]	0 [0–0]	<0.001
Suturing	2 [0–2]	0 [0–0]	<0.001
Cervical examination	0 [0–2]	0 [−1 to 0]	<0.001
CTG	2 [1–2]	0 [0–0]	<0.001
Physiological birth	1 [1–2]	0 [0–0]	<0.001
Pathological birth	2 [1–2]	0 [0–0]	<0.001
OB/GYN skills overall	10 [8–12.5]	0 [−1.5–1]	<0.001

Comparison of differences between self- and objective assessed skills shown as median and interquartile range [Q25–Q75], and significance level. Positive and negative ranks, as well as Z-statistics are omitted for clarity; details are available upon request.

**FIGURE 4:**
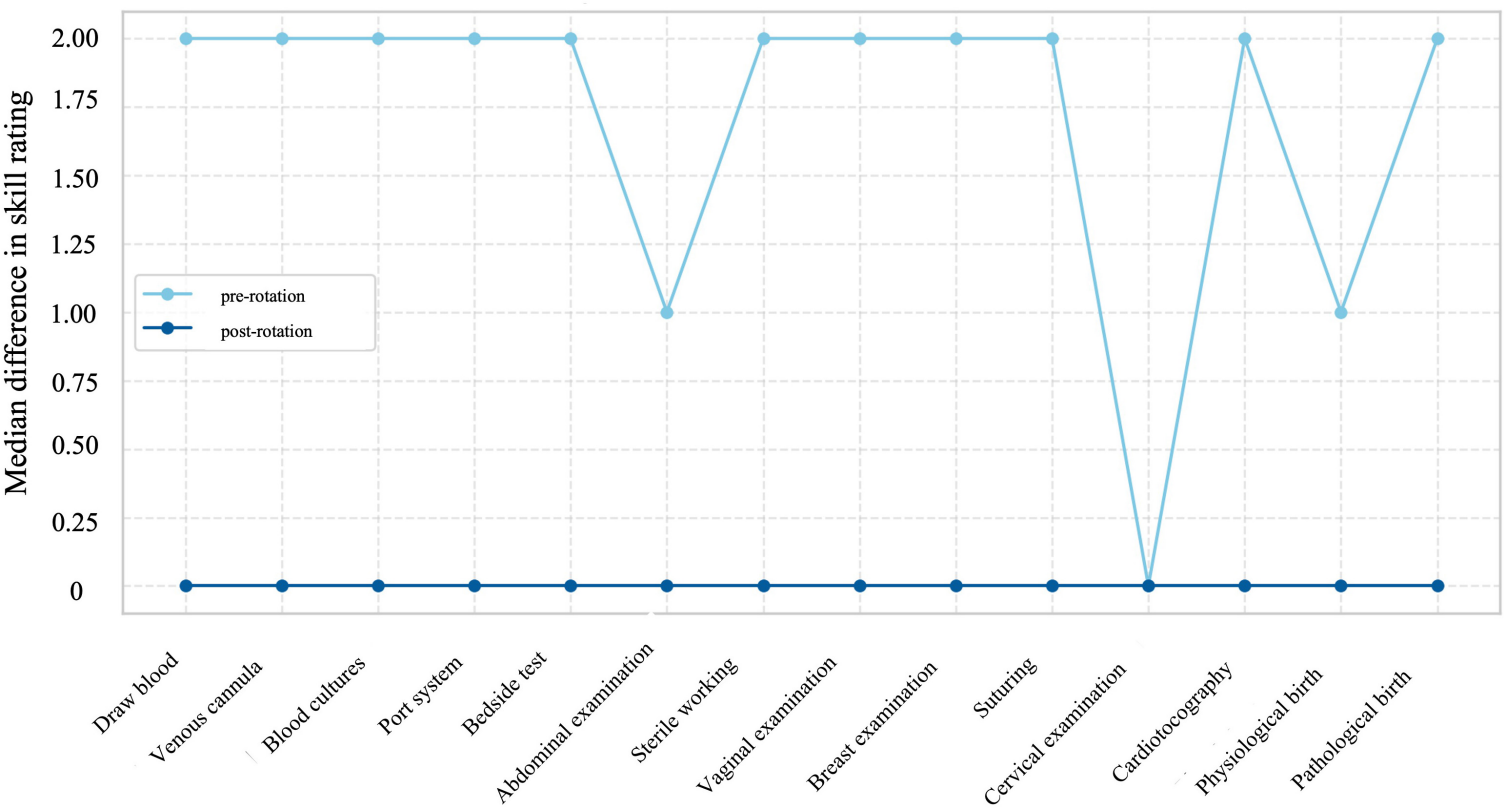
Line plot of discrepancy between self- and objective assessment score, comparing pre- and post-interventional results, showing median difference in rating for each item.

The median discrepancy for general clinical skills decreased from 14 [IQR 9–15] at baseline to 0 [IQR 0–1.5] post-intervention (*Z* = −6.96; *p* < 0.001), and for gynecological skills from 10 [IQR 8–12.5] to 0 [IQR −1 to 5.1]; *Z* = −7.02; *p* < 0.001, as shown in Figure 4.

To explore the relationship between perceived and actual clinical competence, Spearman’s rank correlations were calculated for the 14 skills assessed both subjectively and objectively.

Before the course, a very strong positive correlation was observed between self-assessed and objectively measured scores (ρ = 0.83, *p* < 0.001, *N* = 65). After completion of the *ready4gyn* curriculum, this strong association persisted (ρ = 0.81, *p* < 0.001, *N* = 65).

Students who rated their own skills higher also achieved higher objective performance scores both, before and after the course, indicating a robust alignment between perceived and actual competence throughout the intervention period.

### 3.4 Correlation with clinical rotation order

**FIGURE 5:**
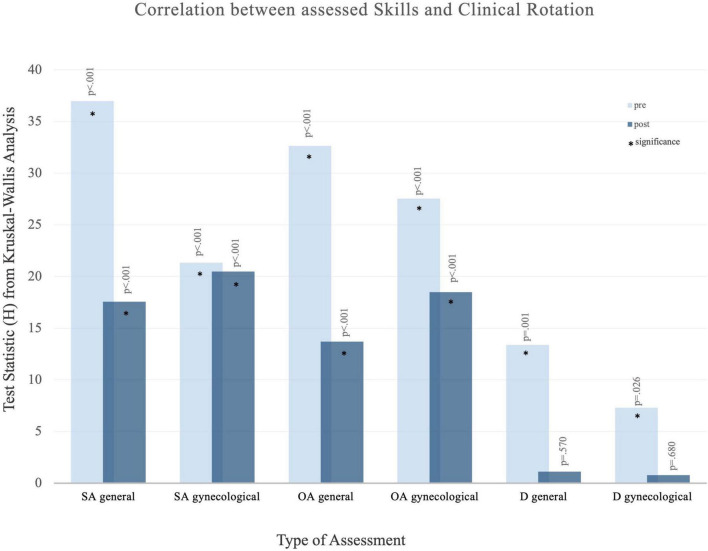
Results of the Kruskal–Wallis test examining the association between assessed clinical skills and clinical rotation (1st, 2nd, 3rd). The *X*-axis shows the type of assessment: SA, self-assessment; OA, objective assessment; D, discrepancy between them. The *Y*-axis displays the H statistic; higher values indicate greater differences between groups.

Within the intervention group (*N* = 65), Kruskal-Wallis tests were used to examine associations between rotation period (first, second, third) and assessment scores (ordinal data). Results are shown in Figure 5. Additional detailed statistical results, including full test statistics and item-level data for all non-parametric analyses, are provided in the Supplementary Table 2.

Significant differences were observed for overall general skills and overall gynecological skills in the self-assessment of students before and after their respective rotation (1 vs. 2 vs. 3), based on the specific tertial (*p* < 0.001) with low scores for first rotation and high scores for third tertial. Before the rotation, 15 of 19 assessed items showed a significant correlation, compared with 10 of 19 items after the rotation.

In addition, a significant difference for both overall general and overall gynecological skills in the objective assessment of students before and after their respective rotation (1 vs. 2 vs. 3), based on the specific rotation period (*p* < 0.001), again with lower scores in the first rotation and higher scores in the third rotation. Before the rotation, 10 of 14 items were significant compared with 6 of 14 items after the rotation.

Differences in self- vs. objective assessments showed significant results prerotation (7 of 14 items), and no significant differences afterward (14 of 14 items non-significant).

No further subgroup analyses (e.g., by age, gender or native language) were conducted due to insufficient variance.

### 3.5 Participant satisfaction and perceived educational value

The training was rated very positively across all categories, with median ratings of 7 (maximum possible score) for key aspects such as professional supervision, clarity of learning objectives, applicability of content to clinical practice, and overall course evaluation (see Table 5). Participants also reported a high learning gain and felt that their interest was stimulated. The opportunity to ask questions and the time frame of the training were also rated highly, though the latter showed slightly greater variability (median = 6 [IQR 5–7]).

**TABLE 5:** Results of the ready4gyn evaluation.

	Intervention group *N* = 65 Median [IQR]
Professional supervision	7 [7–7]
Time frame appropriate	6 [5–7]
Opportunity to ask questions	7 [6–7]
Learning objectives clearly structured	7 [6–7]
Training content applicable to clinical practice	7 [6–7]
High learning gain	7 [7–7]
Interest stimulated	7 [6–7]
Overall evaluation of the ready4gyn course	7 [7–7]

In the free-text responses regarding suggestions and comments, participants expressed a desire for additional course content such as simulation-based ultrasound training, model-based practice of perineal suturing, and training in camera navigation during laparoscopy.

### 3.6 Intervention group (university hospital) vs. comparison group (teaching hospital)

A comparison group consisting of 68 students from affiliated teaching hospitals completed the self-assessment only at the end of their OB/GYN rotation, without prerotation or objective assessments.

Mann-Whitney U tests showed that intervention students reported higher end-of-rotation self-assessed competence across all 19 items compared with students in usual rotations (*p* < 0.001; see Table 6). Additional detailed statistical results, including full test statistics and item-level data for all non-parametric analyses, are provided in the Supplementary Table 3.

**TABLE 6:** Mann-Whitney U test results for comparison of intervention group and control group.

	Intervention group *N* = 65 Median [IQR]	Control group *N* = 68 Median [IQR]	*p*-value
Draw blood	7 [7–7]	6 [5–7]	<0.001
Venous cannula	7 [7–7]	5.5 [4–6]	<0.001
Blood cultures	7 [7–7]	4 [3–5]	<0.001
Port system	7 [6–7]	4 [2–5]	<0.001
Bedside test	7 [6–7]	5 [4–6]	<0.001
Medical history	7 [6–7]	5.5 [5–6]	<0.001
Abdominal examination	7 [6–7]	5 [4–5]	<0.001
Steril working	6 [6–7]	4 [4–5]	<0.001
General skills overall (8 items)	54 [51.5–56]	39 [31.25–44]	<0.001
Vaginal examination	5 [5–5]	1 [1–2]	<0.001
Breast examination	6 [6–7]	4 [3–4.75]	<0.001
Sonography	5 [5–6]	2 [2–3]	<0.001
Suturing	6 [5.5–7]	3 [2.25–4]	<0.001
Laparoscopy	5 [4–6]	2 [1–2]	<0.001
Cervical examination	4 [4–4.5]	1 [1–1]	<0.001
Maternity record	7 [6–7]	5 [4–5]	<0.001
CTG	6 [5–6]	4 [4–5]	<0.001
Physiological birth	6 [5–7]	4 [4–5]	<0.001
Pathological birth	6 [5–6]	3 [2–3]	<0.001
Gynecological skills overall (10 items)	56 [49.5–59]	30 [25–33]	<0.001
Ready for the job	6 [6–7]	4 [3–4]	<0.001

Results of post-rotation self-assessed skills: median, interquartile range [Q25–Q75] and significance level. Mean ranks, as well *U*-values and *Z*-values are omitted for clarity; details are available upon request.

**FIGURE 6:**
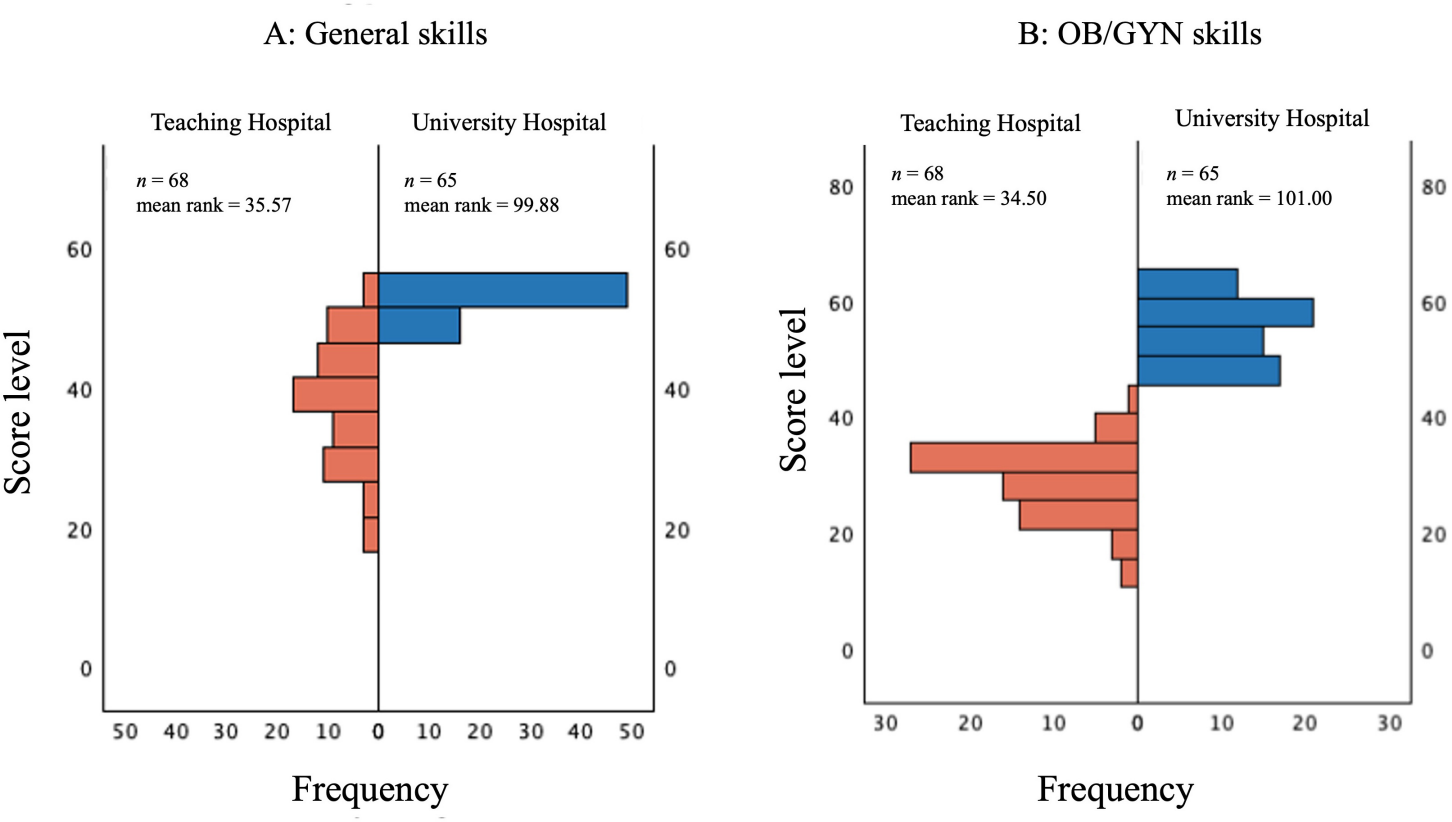
Mann-Whitney U Test results for post-rotation self-assessment of general skills **(A)** and OB/GYN skills **(B)** comparing students of the teaching hospital (control group, *N* = 68) with university hospital (intervention group, *N* = 65). The *Y*-axis shows score levels; the *X*-axis indicates frequency; bars are mirrored for comparison.

For overall general and gynecological skills, participants in the course had higher ranked scores (mean rank = 99.88 and 101, respectively) compared with those who did not attend (mean rank = 35.57 and 34.5, respectively). For general skills, *U* = 73, *Z* = −9.65 and *p* < 0.001 and for gynecological skills, *U* = 0, *Z* = –9.96, *p* < 0.001 for gynecological skills (see Figure 6).

## 4 Discussion

In this study, we evaluated the impact of *ready4gyn*, a simulation-based skills curriculum, on final-year medical students’ self-assessment, objectively measured competence, and the alignment between these two measures during their OB/GYN rotation. Our findings demonstrate that a structured, simulation-based training program was associated with improved confidence, objectively measured performance and self-awareness regarding their skill levels.

This is the first study in Germany to systematically evaluate a simulation-based practical skills curriculum for final-year medical students by combining subjective and objective assessments, and to document a significant improvement in the alignment between self- and objective assessments, reflecting students’ ability to realistically evaluate their own clinical competence.

Our sample included 133 final-year students (65 intervention group, 68 comparison group) with a median age of 26 years and a predominance of female participants (90.2%). Language backgrounds were diverse—85.7% were unilingual native German speakers, with small proportions reported Turkish, Russian, Arabic, Romanian, Persian, and Spanish as their first language—yet German proficiency was uniformly high. The demographic characteristics of both groups were well balanced and mirror typical German medical cohorts (14), supporting the generalizability of our results and reducing the likelihood that confounding factors such as age or language proficiency biased the observed effects. This applies to all reported characteristics except for gender: The proportion of female participants, however, is notably higher than the overall percentage of female medical students in Germany, which is approximately 60% (15). This gender imbalance reflects the typical distribution in OB/GYN electives but may nonetheless limit the generalizability of the findings.

Prior studies have shown that female students tend to underestimate their clinical performance compared with male peers, particularly in low-stakes OSCEs (16, 17). It is therefore conceivable that gender composition influenced self-assessment tendencies in our sample. In addition, the study design was monocentric and relied on voluntary participation in the elective OB/GYN rotation, which may have introduced self-selection bias. Students choosing the university hospital for their rotation may differ from those in teaching hospitals in motivation, prior experience, or expectations toward structured teaching. These factors could partly explain observed differences between groups and should be considered when interpreting the findings. Future multicenter studies with a more balanced gender distribution and randomized group allocation are needed to confirm the generalizability of the results.

Following the four-session *ready4gyn* intervention, students’ self-assessment scores improved significantly across all domains (all *p* < 0.001). Median scores increased from 50 to 54 for general skills and from 41 to 56 for OB/GYN-specific skills. Perceived readiness for clinical work job also improved from 5 to 6 on 7-point Likert scale. Most postrotation medians reached the maximum score of 7, with perfect self-ratings on basic procedures such as drawing blood and venous cannulation. These results suggest that deliberate, hands-on practice in a low-stakes environment substantially enhances students’ self-confidence in performing core clinical tasks.

Objective assessments by trained examiners confirmed these improvements. Median general skills scores increased from 27 to 47 and OB/GYN scores from 17 to 38 (all *p* < 0.001). Importantly, all students demonstrated positive rank changes in the overall objective scores, with no declines observed.

The strong alignment between subjective and objective improvements illustrates that *ready4gyn* does not merely boost confidence, but results in tangible performance gains.

These findings are in line with previous research, such as the study by Kodikara et al., which demonstrated that self-assessed confidence and competence in venipuncture significantly improved immediately after simulation-based training and remained elevated even 1 year later (18). However, most existing studies tend to focus on a single skill or isolated item. In contrast, our study comprehensively assessed 19 different clinical skills and linked self-assessment with objective performance data. Moreover, to our knowledge, this is the first study to systematically investigate the impact of a simulation-based training program on both perceived and objectively measured competence specifically in the context of gynecological and obstetric clinical skills. This broader scope and methodological integration make *ready4gyn* a unique contribution to the field, highlighting the added value of structured, simulation-based curricula in undergraduate medical education.

A key objective of the course was to reduce the gap between perceived and actual performance. We therefore calculated the difference between self-assessed and objectively measured scores pre- and post-intervention. Strikingly, the intervention markedly reduced the discrepancy between self and objective ratings. Median differences fell from 14 to 0 for general and from 10 to 0 for OB/GYN skills (all *p* < 0.001). This recalibration — from systematic overestimation toward better alignment between perceived and actual performance— is highly relevant for patient safety and lifelong learning, as it fosters appropriate humility and the readiness to seek supervision when needed.

It should be noted that the discrepancy score approach (self-assessment minus objective score) may not fully capture the true agreement between perceived and actual performance. A reduction in discrepancy can occur if both measures improve in parallel, without necessarily reflecting a more accurate self-evaluation. Future studies should therefore incorporate correlation analyses or alternative metrics to assess self-assessment alignment more precisely.

The correlation analyses conducted in this study support these considerations. A very strong positive association was observed between self-assessed and objectively measured competence both before (ρ = 0.83, *p* < 0.001) and after the intervention (ρ = 0.81, *p* < 0.001). These findings indicate that students’ self-assessments were already closely aligned with their actual performance and remained closely aligned after the intervention. The consistently high correlation suggests that the reduction in discrepancy scores primarily reflects parallel improvement in both self-assessed and objective performance rather than a measurement artifact. This stable alignment underscores the potential of simulation-based teaching to foster both competency development and realistic self-evaluation.

These findings contrast with those by Madrazo et al. and Jünger et al., who reported that third-year medical students tended to underrate themselves compared with external assessments (16, 17). Particularly, female students were more likely to underestimate their competence. This gender-specific trend could not be confirmed in our predominantly female cohort. Although a formal subgroup analysis was not possible due to limited variation, our results suggest that the structured and empowering learning environment created by *ready4gyn* may counteract such tendencies.

The results also align with research by Schiekirka et al. (19), who demonstrated that aggregated student self-assessments can be a valid indicator of actual learning gains when matched with clearly defined learning objectives. However, they noted that individual-level agreement was limited. This supports the notion that reliable self-evaluation requires structured contexts and continuous calibration—both elements explicitly addressed in the *ready4gyn* curriculum.

Further, Colbert-Getz et al., who reported a weak negative correlation between students’ predicted scores and actual OSCE scores (20), while Davis et al. demonstrated across multiple studies that physicians consistently show limited accuracy in self-assessment, particularly among the least skilled (21). These findings highlight the limits of self-evaluation in the absence of structured feedback and external validation. Deveze et al. reinforced that even in surgical training, accurate self-assessment does not automatically correlate with improved performance (22).

These results further support the notion that improving clinical skills and fostering realistic self-appraisal are distinct but complementary processes, both of which require deliberate instructional design. Our study demonstrates that simulation-based training such as *ready4gyn* can achieve both aims simultaneously by providing structured, objective feedback alongside guided self-reflection. This aligns with findings from Hattie’s synthesis of over 800 meta-analyses on factors influencing student achievement, in which feedback and self-assessment rank among the most powerful instructional strategies (6). By explicitly integrating these high-impact teaching elements into clinical training, simulation-based curricula can enhance both performance and metacognitive accuracy—two outcomes essential for safe and competent clinical practice.

Recent literature highlights the effectiveness of integrated and multimodal educational formats that combine simulation with other evidence-based teaching strategies, such as the flipped classroom approach. For instance, Agostino et al. (23) implemented a flipped classroom–simulation model that significantly improved students’ perceived competence, confidence, and satisfaction in clinical skills training (23). Similar to our findings, their results underline that the integration of preparatory self-directed learning with hands-on, simulation-based practice can enhance not only technical skill acquisition but also self-awareness and reflective learning. In this sense, the *ready4gyn* curriculum represents a comparable multimodal approach within the German undergraduate context, as it systematically combines predefined learning objectives, guided feedback, and experiential simulation sessions. Aligning with current evidence, our results further confirm that simulation-based interventions achieve their greatest educational impact when embedded in structured, competency-oriented teaching frameworks. These observations are further substantiated by the longitudinal study by Stirling et al., who examined the impact of a ward simulation exercise on the development of self-knowledge—a construct encompassing self-awareness, self-efficacy, and self-perception—among final-year medical students (24). Over a 5-year period, the authors demonstrated that simulation can both enhance and, under certain hierarchical assessment conditions, inhibit the development of reflective self-understanding. They emphasized that simulation-based learning achieves its full educational potential when students are not merely assessed but actively engaged as co-participants in the design and reflection process. This perspective aligns with the *ready4gyn* curriculum, which deliberately integrates guided debriefing, formative feedback, and opportunities for self-evaluation to promote not only technical proficiency but also realistic and reflective self-appraisal. In doing so, our approach contributes to the growing body of evidence supporting integrated, learner-centered simulation models that foster both competence and metacognitive awareness in clinical education.

While the intervention group demonstrated substantial pre–post improvements, the comparison group—who completed only end-of-rotation self-assessments—reported lower median competence levels across all 19 items. These between-group differences refer exclusively to postrotation self-assessment and must be interpreted cautiously, as no baseline or objective data were available for the comparison group. Because the study followed a non-randomized design, these between-group differences cannot be interpreted as causal. Differences between groups may also reflect unmeasured factors such as prior experience, motivation, or institutional learning environments, and should not be interpreted as differences in learning gain. The results therefore indicate an association between participation in the structured curriculum and higher end-of-rotation self-assessed competence, without allowing conclusions about causal effects or differential learning gains.

Nevertheless, these results reflect broader challenges and opportunities in postgraduate medical education across German-speaking countries (25). A recent survey of 422 OB/GYN trainees from Germany, Austria, and Switzerland identified revealed systemic issues, including high administrative workloads and limited procedural training opportunities. Notably, trainees who had access to simulation-based education reported feeling “confident to very confident” in performing standard procedures up to 12% more often than those without such training. Practical training in OB/GYN remains insufficient in both quality and quantity. Duhm et al. emphasized the urgent need for reforms to counteract deterrents and improve recruitment into this essential specialty (26).

Meyer et al. further reported that teaching coordinators struggle to balance clinical, research, and teaching demands—often without institutional support—leading to superficial competency-based education and gaps in hands-on skills training (7). In many departments, advanced simulation models are rarely available, and practical training remains limited—particularly in sensitive areas such as gynecological examinations.

Similarly, Larios et al. assessed clinical competence in obstetrics and gynecology using OSCEs (9). Students achieved relatively low overall scores, underscoring the persistent gap between theoretical knowledge and practical clinical skills. Their findings emphasized that written exams alone are insufficient for evaluating clinical competence and that students’ performance is more closely linked to their experience with specific clinical scenarios. These insights further support the implementation of structured, simulation-based training—such as the *ready4gyn* curriculum—to enhance both practical competence and the accuracy of self-assessment.

In contrast to these studies, our work provides a more comprehensive and methodologically robust analysis by combining subjective and objective assessments and evaluating changes over time. It is the first study in Germany to longitudinally demonstrate that a targeted, simulation-based curriculum was associated with higher clinical performance scores and fostered a closer alignment between students’ self-perception and their objectively measured competence. This dual advancement in competence and self-awareness represents an important contribution to the design and evaluation of undergraduate medical education interventions.

During the final year of medical school (PJ), students rotate through different clinical departments, progressively gaining more experience and responsibility. Our findings demonstrate a clear progression in clinical competence and self-assessment across these rotations. Both subjective and objective measures improved significantly with each successive rotation period (all *p* < 0.001), reflecting the expected learning curve during clinical training. The largest differences were observed between students in their first and third tertials. After the rotation, the number of significantly differing items decreased, indicating a growing alignment between perceived and actual skills.

Importantly, the simulation-based training (*ready4gyn*) appeared particularly effective in narrowing performance gaps at the beginning of the practical year. These findings suggest that while clinical exposure naturally fosters competence and self-awareness, targeted simulation training can accelerate this development—especially in the early stages of clinical education.

In addition to measurable improvements, course evaluations reflected high acceptance and perceived relevance of the *ready4gyn* curriculum. Students rated the course highly across all domains, with median ratings of 7 (maximum).

Free-text comments highlighted a desire for additional simulation-based training in ultrasound, laparoscopic camera navigation, and perineal suturing. Previous studies have demonstrated that these skills can be effectively improved through simulation-based training (27–30). The expressed wish for comprehensive ultrasound education via simulation aligns with findings from a nationwide survey conducted in 2019 (31). Approaches to implement simulation-based ultrasound training have shown good results (32).

Since all of these components are essential for daily clinical practice in obstetrics and gynecology, expanding the course to include additional sessions appears to be a meaningful enhancement.

Our findings demonstrate a progressive improvement in clinical competence and self-assessment across final-year medical students, with both subjective and objective measures correlating positively with the stage of clinical rotation (all *p* < 0.001). The simulation-based training appeared particularly effective in narrowing competence gaps in earlier tertials, suggesting that structured simulation can accelerate skill acquisition and self-awareness early in clinical training.

However, limitations of this study should also be reflected upon. The single-center design and the non-randomized group assignment limit the internal validity of the findings. Nevertheless, the high internal consistency of the self-assessment instrument and expert review of the checklists support the reliability and content validity of the applied measures.

As the study followed a quasi-experimental, non-randomized design, differences between university and teaching hospitals may have acted as potential confounders. Factors such as student motivation, supervision quality, or institutional learning culture could have influenced the observed associations. These aspects should be considered when interpreting the results and warrant further investigation in future multicenter or randomized studies.

The study involved a large number of statistical tests across multiple self-assessment, objective, and discrepancy items. Although this increases the risk of type I error, the consistent direction and magnitude of the results, along with the large effect sizes for the aggregated scores, support the robustness of the findings. Nevertheless, item-level results should be interpreted with caution, and future studies may consider formal correction procedures or predefined primary endpoints.

Additionally, the study focused on the elective OB/GYN rotation during the final year of medical training, which is subject to students’ individual preferences. This self-selection may limit the generalizability of the results. Furthermore, the long-term retention of acquired skills and their transferability to actual patient care remain unexamined.

This study should be interpreted in light of its quasi-experimental design, which limits causal inference. Despite these limitations, the pre-and post-comparison and inclusion of a control group provide strong evidence for the observed educational effects.

Future studies should consider randomized, multicenter designs and longitudinal assessments to evaluate the durability of both competence and the accuracy of self-assessment. Moreover, future research should include larger and more gender-balanced samples to examine potential sex-related differences in self-assessment alignment and performance outcomes.

These findings underscore the importance of integrating structured, simulation-based skills training not only during residency but already at the undergraduate level. Early exposure to such programs helps medical students build the confidence, competence, and realistic self-perception required for effective and independent clinical practice. Particularly in a sensitive field such as OB/GYN, simulation offers a valuable and protected environment for learning and refining hands-on skills. The consistently high student ratings further highlight the educational value, practical relevance, and strong acceptance of the curriculum, supporting its feasibility and scalability across other medical faculties. In the long term, these educational strategies may help bridge persistent training gaps in postgraduate education and improve overall preparedness for clinical work.

The *ready4gyn* curriculum—a structured, simulation-based series of four practical sessions—was associated with significant pre-post gains in both perceived and objectively measured competence within the intervention group, and with higher end-of-rotation self-assessed competence compared with students in standard clinical rotations. The median discrepancy between self and objective assessment decreased to near zero post-course (*p* < 0.001), suggesting an improved alignment between perceived and actual competence. Because objective and longitudinal data were collected only in the intervention group, between-group conclusions must be interpreted cautiously and reflect associations rather than causal effects.

The median discrepancy between self and objective assessment decreased to near zero post-course (*p* < 0.001), suggesting an improved alignment between perceived and actual competence.

These benefits were consistent across all tertials within the intervention group. When comparing groups, higher end-of-rotation self-assessed competence was observed among students who participated in the curriculum. Because the comparison group completed only postrotation self-assessments and no baseline or objective assessments, these findings reflect associations rather than differences in learning gain. The median discrepancy between self- and objective assessment decreased to near zero post-course (*p* < 0.001), indicating a closer alignment between perceived and actual competence within the intervention group. Taken together, the findings suggest that integrating regular, focused, hands-on modules into the final-year (Praktisches Jahr) curriculum could help address existing gaps in practical preparation and reflective practice.

To date, it remains largely unknown where and how often structured, simulation-based practical curricula for final-year medical students are implemented—especially in a form that combines systematic self- and objective assessment. The present study represents not only the first comprehensive scientific evaluation of such a curriculum but also the first of its kind in the field of obstetrics and gynecology. As such, it provides unique, much-needed insights into the feasibility, effectiveness, and educational value of simulation-based training in this particularly sensitive and practically demanding specialty.

Future research should evaluate the durability of these gains over time, their transferability to real-world patient care, and the impact on patient outcomes. Additionally, multicenter trials and randomized designs would further validate the generalizability of this training model. Beyond its practical benefits, simulation-based skills training such as *ready4gyn* may also serve as a strategic tool for operationalizing key competencies outlined in the National Competence-Based Catalogue of Learning Objectives for Medicine (NKLM), particularly in the domains of clinical proficiency, self-assessment, and professional development. By fostering both technical competence and guided self-reflection, such training contributes to the development of the “reflective practitioner”—a central ideal in modern medical education aimed at promoting lifelong learning, critical thinking, and responsible clinical decision-making.

## Data Availability

The raw data supporting the conclusions of this article will be made available by the authors, without undue reservation.

## References

[1] Masterplan Medizin. *Standing Conference of the Ministers of Education and Cultural Affairs.* (2020). Available online at: https://www.kmk.org/fileadmin/Dateien/pdf/PresseUndAktuelles/2017/170331_Masterplan_Beschlusstext.pdf (accessed April 9, 2025).

[2] Charokar K Modi J. Simulation-based structured training for developing laparoscopy skills in general surgery and obstetrics and gynecology postgraduates. *J Educ Health Promot.* (2021) 10:48. 10.4103/jehp.jehp_48_21 34912923 PMC8641715

[3] Rosen K. The history of medical simulation. *J Crit Care.* (2008) 23:157–66. 10.1016/j.jcrc.2007.12.004 18538206

[4] Weiss T Rentea R. *Simulation Training and Skill Assessment in Obstetrics and Gynecology.* Treasure Island, TL: StatPearls (2025). 32809343

[5] Zhang M Cheng X Xu A Luo L Yang X. Clinical simulation training improves the clinical performance of Chinese medical students. *Med Educ Online.* (2015) 20:28796. 10.3402/meo.v20.28796 26478142 PMC4609652

[6] Visible Learning. *Hattie-Rangliste: Lehr-Lern-Methoden.* (2025). Available online at: https://visible-learning.org/de/hattie-rangliste-einflussgroessen-effekte-lernerfolg/hattie-rangliste-einfluss-von-lehr-und-lernmethoden/ (accessed April 9, 2025).

[7] Meyer B Riedel F Amann N Graf A Stuehrenberg A Ritter V Exploring the current state of clinical and practical teaching in obstetrics and gynecology in the era of competency-based education: a nationwide survey among German teaching coordinators. *BMC Med Educ.* (2024) 24:165. 10.1186/s12909-024-05138-2 38383443 PMC10880315

[8] Nationaler Kompetenzbasierter Lernzielkatalog Medizin (NKLM). (2025). Available online at: https://nklm.de/zend/menu (accessed May 2, 2025).

[9] Larios Mendoza H Trejo Mejía J Gaviño Ambriz S Cortés Gutiérrez M. Clinical competence evaluation in undergraduate gynecology and obstetrics. *Ginecol Obstet Mex.* (2002) 70:558–65. 12561706

[10] Pierre R Wierenga A Barton M Thame K Branday J Christie C. Student self-assessment in a paediatric objective structured clinical examination. *West Indian Med J.* (2005) 54:144–8. 10.1590/s0043-31442005000200012 15999887

[11] Blanch-Hartigan D. Medical students’ self-assessment of performance: results from three meta-analyses. *Patient Educ Couns.* (2011) 84:3–9. 10.1016/j.pec.2010.06.037 20708898

[12] Gabbard T Romanelli F. Accuracy of health professions students’ self-assessments compared to objective measures of competence. *Am J Pharm Educ.* (2021) 85:8405. 10.5688/ajpe8405 34283796 PMC8086612

[13] Vassiliou M Dunkin B Marks J Fried GM. FLS and FES: comprehensive models of training and assessment. *Surg Clin North Am.* (2010) 90:535–58. 10.1016/j.suc.2010.02.012 20497825

[14] Statistisches Bundesamt. *GENESIS Database.* (2025). Available online at: https://www-genesis.destatis.de (accessed April 11, 2025).

[15] Medizinische Fakultät der Universität zu Köln. *Gender-Datenreport.* (2024). Available online at: https://medfak.uni-koeln.de/sites/MedFakDekanat/Akademische_Entwicklung_Gender/Gender-Datenreport_2024.pdf (accessed January 15, 2025).

[16] Jünger J Schellberg D Nikendei C. Subjektive Kompetenzeinschätzung von Studierenden und ihre Leistung im OSCE. *GMS Z Med Ausbild.* (2006) 23:Doc51.

[17] Madrazo L Lee C McConnell M Khamisa K. Self-assessment differences between genders in a low-stakes OSCE. *BMC Res Notes.* (2018) 11:393. 10.1186/s13104-018-3494-3 29903050 PMC6003209

[18] Kodikara K Seneviratne T Premaratna R. Pre-clerkship procedural training in venipuncture: a prospective cohort study on skills acquisition and durability. *BMC Med Educ.* (2023) 23:729. 10.1186/s12909-023-04722-2 37803328 PMC10559527

[19] Schiekirka S Reinhardt D Beißbarth T Anders S Pukrop T Raupach T. Estimating learning outcomes from pre- and posttest student self-assessments: a longitudinal study. *Acad Med.* (2013) 88:369–75. 10.1097/ACM.0b013e318280a6f6 23348083

[20] Colbert-Getz J Fleishman C Jung J Shilkofski N. How do gender and anxiety affect students’ self-assessment and actual performance on a high-stakes clinical skills examination? *Acad Med.* (2013) 88:44–8. 10.1097/ACM.0b013e318276bcc4 23165273

[21] Davis D Mazmanian P Fordis M Van Harrison R Thorpe K Perrier L. Accuracy of physician self-assessment compared with observed measures of competence: a systematic review. *JAMA.* (2006) 296:1094–102. 10.1001/jama.296.9.1094 16954489

[22] Deveze E Traore A Ribault N Estoppey D Latelise B Fournier H Self-assessment versus peer-assessment in microsurgery learning: a retrospective study. *J Surg Educ.* (2023) 80:1472–8. 10.1016/j.jsurg.2023.06.028 37524617

[23] Agostino S Cherasco GM Papotti G Milan A Abate Daga F Abate Daga M Impact of simulation-based and flipped classroom learning on self-perceived clinical skills compared with traditional training. *Educ Sci.* (2025) 15:31. 10.3390/educsci15010031

[24] Stirling K Roger A Topping K. Does simulation in medical education enhance or inhibit the development of self-knowledge? *J Appl Learn Teach.* (2024) 6:S1–11. 10.37074/jalt.2023.6.S1.11

[25] Winder F Breuer G Favero M Foessleitner P Friemann M Krischer B Postgraduate medical education in obstetrics and gynaecology: where are we now? *GMS J Med Educ.* (2022) 39:Doc41. 10.3205/zma001562 36310887 PMC9585411

[26] Duhm L Wittek A Plöger R Haverkamp N Marinova M Strizek B Exploring undergraduate medical students’ perceptions and career choices in obstetrics and gynecology. *Geburtshilfe Frauenheilkd.* (2025) 85:333–43. 10.1055/a-2500-0078 40052015 PMC11882317

[27] Cook J Rao V Bell F Durkin M Cone J Lane-Cordova A Simulation-based learning using ultrasound for OB/GYN scenarios. *J Clin Ultrasound.* (2020) 48:457–61. 10.1002/jcu.22888 32691423

[28] Hartup L Lewis A Medrano G. Transvaginal ultrasound simulation: educational benefits on obstetrics and gynecology clerkship. *Clin Teach.* (2025) 22:e70019. 10.1111/tct.70019 39789799

[29] Olmes G Doerk M Solomayer E Nigdelis M Sima R Hamoud B. Assessment of students’ technical skills in second-degree perineal repair using sponge-based simulation. *Arch Gynecol Obstet.* (2024) 310:893–7. 10.1007/s00404-023-07297-x 38081960 PMC11258089

[30] Nilsson C Sorensen J Konge L Westen M Stadeager M Ottesen B Simulation-based camera navigation training in laparoscopy. *Surg Endosc.* (2017) 31:2131–9. 10.1007/s00464-016-5210-5 27770252 PMC5411407

[31] Recker F Barth G Lo H Haverkamp N Nürnberg D Kravchenko D Students’ perspectives on ultrasound education in German medical schools. *Front Med.* (2021) 8:758255. 10.3389/fmed.2021.758255 34901071 PMC8655332

[32] Weimer J Recker F Hasenburg A Buggenhagen H Karbach K Beer L Development and evaluation of a simulator-based ultrasound training program for university teaching in obstetrics and gynecology. *Front Med.* (2024) 11:1371141. 10.3389/fmed.2024.1371141 38721350 PMC11076731

