# Supplementary material for: Bridging the gap: effects of simulation-based OB/GYN training on skills and self-perception in final-year medical students
Source: Front Med (Lausanne). 2025 Dec 16;12:1716282. doi: 10.3389/fmed.2025.1716282 (PMC12747925; doi:10.3389/fmed.2025.1716282)
Supplement: Supplementary file 1 [file Supplementary_file_1.docx]

**Supplementary Material** „Bridging the Gap: Effects of Simulation-Based OB/GYN Training on Skills and Self-Perception in Final-Year Medical Students”

**Table S1**. Complete Wilcoxon Signed-Rank Test results for changes in pre-/post-intervention comparison

| **Skill** | **Pre-rotation**  *n* = 65  (median [IQR] ) | **Post-rotation**  *n* = 65  (median [IQR] ) | **Positive Ranks** (n) | **Negative Ranks** (n) | **Ties**  (n) | **Z-value** | **p-value** |
| --- | --- | --- | --- | --- | --- | --- | --- |
| Draw blood | 2 [1-3] | 0 [0-0] | 0 | 59 | 6 | -6.76 | <.001 |
| Venous cannula | 2 [2-2] | 0 [0-0] | 0 | 58 | 7 | -6.87 | <.001 |
| Blood cultures | 2 [1-2] | 0 [0-0] | 0 | 61 | 4 | -6.90 | <.001 |
| Port system | 2 [1-3] | 0 [0-0.5] | 0 | 56 | 9 | -6.60 | <.001 |
| Bedside test | 2 [1-2] | 0 [0-0] | 1 | 55 | 9 | -6.54 | <.001 |
| Abdominal examination | 1 [1-2] | 0 [0-0] | 11 | 49 | 5 | -4.99 | <.001 |
| Steril working | 2 [1-3] | 0 [0-0] | 0 | 54 | 11 | -6.46 | <.001 |
| General skills overall | 14 [9-15] | 0 [0-1.5] | 0 | 64 | 1 | -6.96 | <.001 |
| Vaginal examination | 2 [1-3] | 0 [0-1] | 2 | 50 | 12 | -6.16 | <.001 |
| Breast examination | 2 [1-3] | 0 [0-0] | 0 | 65 | 0 | -7.16 | <.001 |
| Suturing | 2 [0-2] | 0 [0-0] | 3 | 50 | 12 | -6.10 | <.001 |
| Cervical examination | 0 [0-2] | 0 [-1-0] | 4 | 45 | 16 | -5.69 | <.001 |
| CTG | 2 [1-2] | 0 [0-0] | 1 | 59 | 5 | -6.76 | <.001 |
| Physiological birth | 1 [1-2] | 0 [0-0] | 3 | 55 | 7 | -5.95 | <.001 |
| Pathological birth | 2 [1-2] | 0 [0-0] | 6 | 52 | 7 | -6.24 | <.001 |
| OB/GYN skills overall | 10 [8-12.50] | 0 [-1.5-1] | 0 | 65 | 0 | -7.02 | <.001 |

Table Legend: Comparison of differences between self- and objective assessed skills shown as median and interquartile range [Q25 - Q75], direction of change, Z-statistics and significance level.

**Table S2.** Complete results of Kruskal-Wallis Test evaluating the correlation between clinical rotation and assessment results.

|  | **Self-assessment pre** | **Self-assessment post** | **Objective assessment pre** | **Objective assessment post** | **Discrepancy pre** | **Discrepancy post** |
| --- | --- | --- | --- | --- | --- | --- |
| Draw blood | 26.84; p<.001 | .000; p=1.000 | 32.16; p<.001 | 1.24; p=.538 | 19.14; p<.001 | 1.24; p=.538 |
| Venous cannula | 37.86; p<.001 | 000; p=1.000 | 30.41; p<.001 | 5.63; p=.060 | 3.25; p=.197 | 5.63; p=.060 |
| Blood cultures | 29.99; p<.001 | 1.21; p=.546 | 27.70; p<.001 | 5.28; p=.071 | 8.0; p=.018 | 3.61; p=.165 |
| Port system | 29.07; p<.001 | 12.77; p=.002 | 24.75; p<.001 | 15.16; p<.001 | 4.11; p=.128 | 9.2; p=.010 |
| Bedside test | 25.60; p<.001 | 15.56; p<.001 | 30.03; p<.001 | 22.26; p<.001 | 12.16; p=.002 | 3.10; p=.216 |
| Medical history | 10.01; p=.007 | 12.95; p=.002 | n.a. | n.a. | n.a. | n.a. |
| Abdominal examination | 12.88; p=.002 | 15.49; p<.001 | 32.39; p<.001 | 7.90; p=.019 | 24.47; p<.001 | 17.90; p<.001 |
| Sterile working | 17.69; p<.001 | 1.27; p=.531 | 19.87; p<.001 | 4.57; p=.102 | 13.60; p=.001 | 5.97; p=.050 |
| General skills overall | 36.99; p<.001 | 17.57; p<.001 | 32.64; p<.001 | 13.71; p<.001 | 13.41; p=.001 | 1.12; p=.570 |
| Vaginal examination | 7.48; p=.024 | 5.29; p=.071 | 5.31; p=.070 | 1.74; p=.419 | 3.67; p=.160 | 3.02; p=.221 |
| Breast examination | 21.97; p<.001 | 8.15; p=.017 | 19.02; p<.001 | 4.79; p=.091 | 4.20; p=.123 | 4.09; p=.129 |
| Sonography | 25.43; p<.001 | 19.38; p<.001 | n.a. | n.a. | n.a. | n.a. |
| Suturing | 11.89; p=.003 | 17.83; p<.001 | 35.89; p<.001 | 17.79; p<.001 | 22.85; p<.001 | .370 ; p=.831 |
| Laparoscopy | 24.25; p<.001 | 32.87; p<.001 | n.a. | n.a. | n.a. | n.a. |
| Cervical examination | 17.00; p<.001 | .439; p=.803 | 2.34; p=.310 | .939; p=.625 | 17.67; p<.001 | .071; p=.965 |
| Maternity record | 10.61; p=.005 | 5.40; p=.067 | n.a. | n.a. | n.a. | n.a. |
| CTG | 5.78; p=.056 | 2.43; p=.296 | 6.15; p=.046 | 9.133; p=.010 | .276; p=.871 | 5.79; p=.055 |
| Physiological birth | 5.53; p=.063 | 14.64; p<.001 | 29.01; p<.001 | 14.32; p<.001 | 13.67; p<.001 | 2.60; p=.272 |
| Pathological birth | 1.64; p=.441 | 13.17; p=.001 | 21.74; p<.001 | 17.31; p<.001 | 22.7; p<.001 | .374; p=.830 |
| OB/GYN overall | 21.34; p<.001 | 20.48; p<.001 | 27.54; p<.001 | 18.48; p<.001 | 7.29; p=.026 | .773; p=.680 |
| Ready for the job | 17.75; p<.001 | 13.0; p=.002 | n.a. | n.a. | n.a. | n.a. |

Table Legend: Results are demonstrated as self-assessment, objective assessment and discrepancy between self- and objective assessment pre- and post-rotation respectively for each item, and reported as H (df=2) and p-value. N.a. indicating not assessed.

**Table S3**. Complete Mann-Whitney U test results for comparison of intervention group and control group.

|  | **Intervention group**  ***N* = 65** | | **Control group *N* =68** | |  |  |  |
| --- | --- | --- | --- | --- | --- | --- | --- |
|  | Mean [IQR] | Mean rank | Mean [IQR] | Mean rank | U-value | Z-value | p-value |
| Draw blood | 7 [7-7] | 89 | 6 [5-7] | 45.97 | 780 | -7.73 | <.001 |
| Venous cannula | 7 [7-7] | 93.5 | 5.5 [4-6] | 41.67 | 487 | -8.79 | <.001 |
| Blood cultures | 7 [7-7] | 98.27 | 4 [3-5] | 37.11 | 177 | -9.6 | <.001 |
| Port system | 7 [6-7] | 99.69 | 4 [2-5] | 35.75 | 85 | -9.8 | <.001 |
| Bedside test | 7 [6-7] | 94.81 | 5 [4-6] | 40.42 | 402 | -8.46 | <.001 |
| Medical history | 7 [6-7] | 91.35 | 5.5 [5-6] | 43.72 | 627 | -7.51 | <.001 |
| Abdominal examination | 7 [6-7] | 95.38 | 5 [4-5] | 39.88 | 365 | -8.6 | <.001 |
| Steril working | 6 [6-7] | 100.29 | 4 [4-5] | 35.18 | 46 | -10.0 | <.001 |
| General skills overall (8 items) | 54 [51.5-56] | 99.88 | 39 [31.25-44] | 35.57 | 73 | -9.65 | <.001 |
| Vaginal examination | 5 [5-5] | 101 | 1 [1-2] | 34.5 | 0 | -10.32 | <.001 |
| Breast examination | 6 [6-7] | 100.48 | 4 [3-4.75] | 35 | 34 | -10.01 | <.001 |
| Sonography | 5 [5-6] | 100.48 | 2 [2-3] | 34.99 | 33 | -9.98 | <.001 |
| Suturing | 6 [5.5-7] | 100.38 | 3 [2.25-4] | 35.09 | 40 | -991 | <.001 |
| Laparoscopy | 5 [4-6] | 100.5 | 2 [1-2] | 34.98 | 32 | -9.96 | <.001 |
| Cervical examination | 4 [4-4.5] | 99.17 | 1 [1-1] | 36.25 | 119 | -9.89 | <.001 |
| Maternity record | 7 [6-7] | 95.07 | 5 [4-5] | 40.17 | 385 | -8.45 | <.001 |
| CTG | 6 [5-6] | 96.6 | 4 [4-5] | 38.71 | 286 | -9.0 | <.001 |
| Physiological birth | 6 [5-7] | 97.42 | 4 [4-5] | 37.93 | 233 | -9.15 | <.001 |
| Pathological birth | 6 [5-6] | 99.92 | 3 [2-3] | 35.54 | 70 | -9.78 | <.001 |
| Gynecological skills overall (10 items) | 56 [49.5-59] | 101 | 30 [25-33] | 34.5 | 0 | -9.96 | <.001 |
| Ready for the job | 6 [6-7] | 99.98 | 4 [3-4] | 35.47 | 66 | -9.85 | <.001 |

Table Legend: Results of post-rotation self-assessed skills: median, interquartile range [Q25-Q75], mean rank, U-value, Z-values and significance level.
